# Supplementary material for: Analysis of PPARγ Signaling Activity in Psoriasis
Source: Int J Mol Sci. 2021 Aug 10;22(16):8603. doi: 10.3390/ijms22168603 (PMC8395241; doi:10.3390/ijms22168603)
Supplement: Supplementary file 1 [file ijms-22-08603-s001.zip › Supplemental materials_Analysis of PPARg signaling activity in psoriasis/Pathway models/Models images and html files/PPARG inhibits phenotypes assotiated with psoriasis/142067.html]

non-selective vesicle exocytosis


# Cell Process non-selective vesicle exocytosis

|  |  |
| --- | --- |
| URN | urn:agi-gocellproc:0006887 |
| Total Entities | 4 |
| Connectivity | 3291 |
| Name | non-selective vesicle exocytosis |

---

|  |  |
| --- | --- |
| ChildConcepts | synaptic vesicle exocytosis |
|  | calcium ion dependent exocytosis |
|  | regulated secretory pathway |
|  | lytic viral exocytosis |

---

|  |  |
| --- | --- |
| Pathway | High Fat Diet Activates cAMP Related Exocytosis in Synapse and Endocrine Cell |
|  | K-cell: GIP Production |
|  | D-cell: Somatostatin Release |
|  | G-cell: Gastrin Secretion |
|  | Serotonin Release |
|  | Dopamine Release in Nociception |
|  | GABA Release |
|  | Acetylcholine Release |
|  | Epinephrine/Norepinephrine Release |
|  | ADORA1/2A -> Exocytosis |
|  | ADRA2A -> Neurotransmitter Release |
|  | CHRNA3-B4/A4-B2/A7 -> Ion Transport |
|  | GABA(B)R -> Postsynaptic Inhibition |
|  | P2RXs -> Synaptic Transmission |
|  | Tight Junction Assembly (Claudins) |
|  | Tight Junction Assembly (JAMs) |
|  | beta-Cell Granules Exocytois |
|  | Natural Killer Cell in Diabetes Mellitus Type 1 |
|  | Leptin in Insulin Synthesis and Secretion |
|  | G-Proteins Signaling in Insulin Secretion in beta-Cell |
|  | beta-Cell Function Inhibition by Ciclosporine and Tacrolimus (Rodent Model) |
|  | Olanzapine Effect on Insulin Secretion in Diabetes Mellitus |
|  | Ghrelin Influence on Insulin Secretion (Hypothesis) |
|  | FOXO1 and SREBP-1C Role in beta-Cell Suppression (Rodent Model) |
|  | Goblet-Cell Exocytosis in Asthma |
|  | Amyloid beta and APP Intracellular Transport in Alzheimer Disease |
|  | Invadopodia Formation in Cancer Cells |
|  | PPARG inhibits phenotypes assotiated with psoriasis |
|  | efects selected |
|  | 3\_1\_Mucus accumulation in asthma |
|  | 1\_2\_Insulin synthesis and secretion: insulin secretion |

---

|  |  |
| --- | --- |
| MedScan ID | 4000005 |

---

|  |  |
| --- | --- |
| Alias | vesicle exocytosis |
|  | externalizations |
|  | externalization |
|  | non selective vesicle exocytoses |
|  | nonselective vesicle exocytosis |
|  | exo-cytosis |
|  | vesicle exocytoses |
|  | exocytosis |
|  | nonselective vesicle exocytoses |
|  | non selective vesicle exocytosis |
|  | exocytotic |
|  | exocytoses |
|  | non-selective vesicle exocytoses |
|  | non-selective vesicle exocytosis |

---

|  |  |
| --- | --- |
| GO ID | 0006887 |

---
